# Ⅰ期非小细胞肺癌术后预后的多因素分析

**DOI:** 10.3779/j.issn.1009-3419.2011.12.05

**Published:** 2011-12-20

**Authors:** 俊 裴, 宝惠 韩, 杰 张, 爱琴 顾

**Affiliations:** 200030 上海，上海交通大学附属上海市胸科医院肺内科 Department of Pulmonary Medicine, Shanghai Chest Hospital Affiliated to Shanghai Jiaotong University, Shanghai 200030, China

**Keywords:** 肺肿瘤, 预后, 生存分析, Lung neoplasms, Prognosis, Survival analysis

## Abstract

**背景与目的:**

Ⅰ期患者尤其是Ⅰb期术后非小细胞肺癌(non-small cell lung cancer, NSCLC)患者能否从辅助化疗中获益备受争议。2009年第7版肺癌TNM分期发表。本研究的目的是明确新版TNM分期对Ⅰ期NSCLC患者的价值, 以及评估术后辅助化疗是否能提高早期肺癌患者的生存率。

**方法:**

研究纳入了上海市胸科医院1998年6月-2010年6月早期NSCLC完全切除术的433例患者, 按照新的分期标准重新分期。把新分期的参数与其它已被证明与肺癌预后相关的因子结合起来, 进行多因素分析。研究纳入变量包括年龄、性别、吸烟史、病理类型、手术切除方式(叶切、双叶切、袖切和全切)、肿瘤大小(肿瘤最长径)、T分期、淋巴管血管癌栓和辅助化疗。

**结果:**

女性患者3年生存率优于男性(89.22% *vs* 77.53%, *P*=0.001, 8)。老年患者预后不佳, ≥70岁的NSCLC患者3年生存率为70.64%, < 70岁为85.85%, *P*=0.000, 1)。肿瘤最长径≤2 cm患者的3年生存率优于 > 2 cm且≤3 cm患者(95.15% *vs* 85.71%), 肿瘤最长径 > 3 cm且≤5 cm及 > 5 cm且≤7 cm者3年生存率为74.80% *vs* 60.47%(*P* < 0.000, 1)。多因素分析显示年龄、性别、血管癌栓、病理类型、胸膜侵犯是影响生存期的预后因素。

**结论:**

对Ⅰ期NSCLC患者而言, 肿瘤最长径及病理类型是独立的预后因素。腺癌患者的生存期优于其它病理类型。女性和非吸烟患者结局较好。Ⅰb期患者可能从术后辅助化疗中获益。

## Abstract (en)

**Background and objective:**

The effectiveness of adjuvant chemotherapy in providing survival advantage for stage Ⅰ non-small cell lung cancer (NSCLC) patients, especially those with stage Ⅰb NSCLC, remains to be determined.The seventh edition of the Tumor Node Metastasis (TNM) Classification of Malignant Tumors is due to be published in 2009.The aim of the current study is to validate the value of this classification in Chinese early-stage NSCLC.The benefits of adjuvant chemotherapy to patients with early-stage NSCLC were also assessed.

**Methods:**

The new staging project was validated in 433 patients who underwent complete surgical resection for early-stage NSCLC at the Single Institution of Shanghai Chest Hospital from June 1998 to June 2010.This new parameter was combined with other well-established prognostic factors, and multivariate survival analysis were performed.Variables in the analysis included age, gender, history of smoking, pathologic type, type of resection (pneumonectomy, lobectomy, bilobectomy, and sleeve resection), tumor size (largest tumor dimension), T-status, lymphovascular vessel invasion, and adjuvant chemotherapy.

**Results:**

The three-year overall survival rates for females and males are 89.22% and 77.53%, respectively (*P*=0.001, 8).Elder patients have worse prognoses:the survival rates for those aged ≥70 and < 70 are 70.64% and 85.85%, respectively (*P*=0.000, 1).The three-year overall survival rates of patients whose tumors measured no larger than 2 cm in biggest diameter or larger than 2 cm but no larger than 3 cm are 95.15% and 85.71%, respectively.For those with tumors larger than 3 cm but smaller than 5 cm or larger than 5 cm but smaller than 7 cm, the survival rates are 74.80% and 60.47%, respectively (*P* < 0.000, 1).Multivariate analysis reveals that age, gender, vascular vessel invasion, pathologic type, and visceral pleural involvement are significant predictive factors of the overall survival.

**Conclusion:**

The tumor size and pathologic type are significant independent prognostic factors in stage Ⅰ NSCLC.The survival rates of patients with adenocarcinoma are higher than those of patients with other types of NSCLC.Female patients and those without a history of smoking have a better outcome.Results suggest that patients with the Ⅰb stage of the disease can benefit from adjuvant chemotherapy.

对早期非小细胞肺癌(non-small cell lung cancer, NSCLC)患者而言, 手术切除是主要治疗手段。但是, 即使进行完全切除术Ⅰ期患者的结局也有差异, 5年生存率为45%-65%^[1, 2]^。2002年后国际上以第六版TNM分期系统作为NSCLC分期的标准, 但Ⅰ期NSCLC患者根治术后生存期的差异清楚地表明该分期标准至少对早期肺癌而言并不充分。因此, 国际肺癌研究联合会(international association for the study of lung cancer, IASLC)收集来自欧洲、亚洲、北美洲和澳大利亚等20多个国家、40多个中心100, 869例肺癌病例的临床资料, 其中有81, 015例满足TNM分期、病理和生存期随访的要求, 包括67, 725例NSCLC和13, 290例小细胞肺癌(small cell lung cancer, SCLC), 就TNM分期与预后之间的相关性做了深入分析^[3, 4]^。2007年世界肺癌大会上提出了对第7版肺癌TNM分期的修改建议, 根据肿瘤大小不同, 将原来的T分期分为T1a(肿瘤最大直径≤2 cm)、T1b(3 cm≥肿瘤最大直径 > 2 cm)、T2a(5 cm≥肿瘤最大直径 > 3 cm)、T2b(7 cm≥肿瘤最大直径 > 5 cm)。本文根据新TNM分期标准, 对上海市胸科医院早期NSCLC术后患者的生存情况进行多因素分析。

## 1 方法

### 1.1 病例资料

对上海市胸科医院1998年6月-2010年6月临床诊断为Ⅰ期或Ⅱ期肺癌并进行完全切除手术的病例资料进行回顾性分析。长期电话随访了474例患者, 其中433例患者可获得生存数据。所有患者经病理确诊为腺癌、鳞癌、腺鳞癌、大细胞癌等, 排除小细胞肺癌。术前评价和分期包括完整的病史采集、体格检查、气管镜、血常规、血清生化检测、胸CT、脑CT、全身骨核素扫描、上腹部B超或PET-CT。

### 1.2 治疗

所有患者进行了叶切、双叶切和全切。切端镜下证实未见癌转移。手术时由手术医师根据患者病变范围和心肺功能储备情况决定肺切除范围和纵隔淋巴结清扫范围。所有手术符合美国国家综合癌症网(national comprehensive cancer network, NCCN)定义的完全性切除标准^[5-8]^。

肺部手术进行系统性淋巴结取样, 所有患者纵隔区取样一个或多个淋巴结。对于右侧肺癌, 行纵隔淋巴结充分清扫时取样2R、4R、7、8、9站淋巴结; 左侧肺癌取样4L、5、6、7、9站淋巴结^[9]^。与纵隔淋巴结取样相比, 完全性纵隔淋巴结清扫术的并发症并不增加^[10]^。

由于辅助化疗对早期NSCLC患者的生存获益存在争议, 化疗的进行与否取决于患者的TNM分期、病理类型、分化程度、欧洲肿瘤协会(european co-operative oncology group, ECOG)评分、年龄、夹杂症等综合因素, 并由胸外科及肿瘤内科医生结合患者意愿、经济情况及耐受性共同决定。局部复发前患者未进行辅助放疗。

### 1.3 统计分析

使用SPSS 11.5统计软件进行统计学分析。整体生存期(overall survival, OS)计算方法为诊断日期(即手术日期)直至死亡日期或存活患者的最终随访日期。无病生存期(disease free survival, DFS)的计算方法为治疗完成直至疾病复发。用*Kaplan-Meier*法计算生存概率。预后分析的变量包括年龄、性别、吸烟史、组织病理学诊断、淋巴管癌栓或血管癌栓的形成、清扫的淋巴结组数和淋巴结个数、手术切除方式(叶切、双叶切、袖切和全切)、肿瘤最长径和T分期。单因素生存分析使用*Log-rank*检验比较每个变量(预后因素)分层后的不同生存概率。根据统计分析结果决定纳入*Cox*回归分析的变量, *P* < 0.05的与生存概率可能相关的因素将被纳入多因素分析的*Cox*回归模型。*P* < 0.05为差异有统计学意义。

## 2 结果

### 2.1 纳入研究的病例资料

共433例病例最终纳入研究, 中位年龄为61岁(29岁-79岁)。手术方式为叶切(365例)、双叶切除(32例)、全肺切除(19例)和袖切(17例)。按照2009年TNM分期标准, 包括55例T1期、347例T2期、31例T3期NXCLC患者。181例患者进行了术后辅助化疗。在Ⅰb期NSCLC患者中, 174例患者病灶最长径≤3 cm但病灶侵犯胸膜。所有组织的石蜡切片由2位病理科医师进行病理组织学诊断, 腺癌比例最高(64.2%)。表 1显示了研究病例的具体临床资料, 包括性别、年龄、吸烟史、组织学类型、手术类型、肿瘤最长径、T分期、TNM分期、辅助化疗、淋巴管血管癌栓、胸膜侵犯等情况。

**1:** 患者特点及临床因素的单因素分析 Patient characteristics and univariate analysis according to clinical factors

Patient characteristics	*n*	3-yr survival rate	*P*
Alive			
Yes	327 (75.5%)	-	
No	106 (24.5%)	-	
Gender			
Male	266 (61.4%)	77.53%	0.001, 8
Female	167 (38.6%)	89.22%	
Age (yr)			
≥70	109 (25.2%)	70.64%	0.000, 1
< 70	324 (74.8%)	85.85%	
Histology			
Adenocarcinoma	278 (64.2%)	87.96%	< 0.000, 1
Squamous cell carcinoma	98 (22.6%)	79.59%	
Adenosquamous cell carcinoma	50 (11.5%)	58.00%	
Large-cell carcinoma	3 (0.7%)	66.67%	
Other types	4 (1.0%)	57.14%	
Smoking status			
≥400	172 (39.7%)	77.59%	0.035, 2
< 400	261 (60.3%)	85.00%	
Greatest dimension of tumor (cm)			
≤2	103 (23.8%)	95.15%	< 0.000, 1
2 < T≤3	126 (29.1%)	85.71%	
3 < T≤5	142 (32.8%)	74.80%	
5 < T≤7	43 (9.9%)	60.47%	
> 7	19 (4.4%)	68.42%	
T stage			
T1a	33 (7.6%)	90.91%	< 0.000, 1
T1b	22 (5.1%)	81.82%	
T2a	309 (71.4%)	85.48%	
T2b	38 (8.8%)	57.89%	
T3	31 (7.1%)	67.74%	
TNM stage			
Ⅰa	55 (12.7%)	90.91%	< 0.000, 1
Ⅰb	309 (71.4%)	81.82%	
Ⅱa	38 (8.8%)	85.48%	
Ⅱb	31 (7.1%)	57.89%	
Type of resection			
Lobectomy	365 (84.3%)	82.51%	0.872, 8
Bilobectomy	32 (7.4%)	81.25%	
Pneumonectomy	19 (4.4%)	100.00%	
Sleeve resection	17 (3.9%)	82.35%	
Lympho invasion			
Yes	11 (2.5%)	63.64%	0.073, 8
No	422 (97.5%)	82.46%	
Vascular invasion			
Yes	8 (1.8%)	62.50%	0.110, 0
No	425 (98.2%)	82.35%	
Adjuvant chemotherapy			
≥1 cycle	181 (41.8%)	87.29%	0.013, 3
No	252 (58.2%)	78.26%	
Visceral pleural involvement			
Yes	331 (76.4%)	81.06%	0.869, 8
No	102 (23.6%)	85.87%	

### 2.2 整体生存期

所有患者术后进行电话随访, 中位随访时间为为59.85个月(14.33个月-136.30个月)。截至2011年8月9日, 共327例(75.5%)患者存活。整组患者达到3年中位生存期, 3年生存率为82%。肿瘤最长径、T分期、TNM分期、性别、年龄、组织学分类、吸烟史、辅助化疗在整体生存的单因素分析中有统计学意义(表 1)。不同的肿瘤最长径患者有生存差异(*P* < 0.000, 1, 图 1), 肿瘤最长径≤2 cm患者的3年生存率为95.15%, 2 cm < 肿瘤最长径≤3 cm患者的3年生存率为85.71%;3 cm < 肿瘤最长径≤5 cm患者的3年生存率为74.80%, 5 cm < 肿瘤最长径≤7 cm患者的3年生存率为60.47%, 肿瘤最长径 > 7 cm患者的3年生存率为68.42%。

**1:**
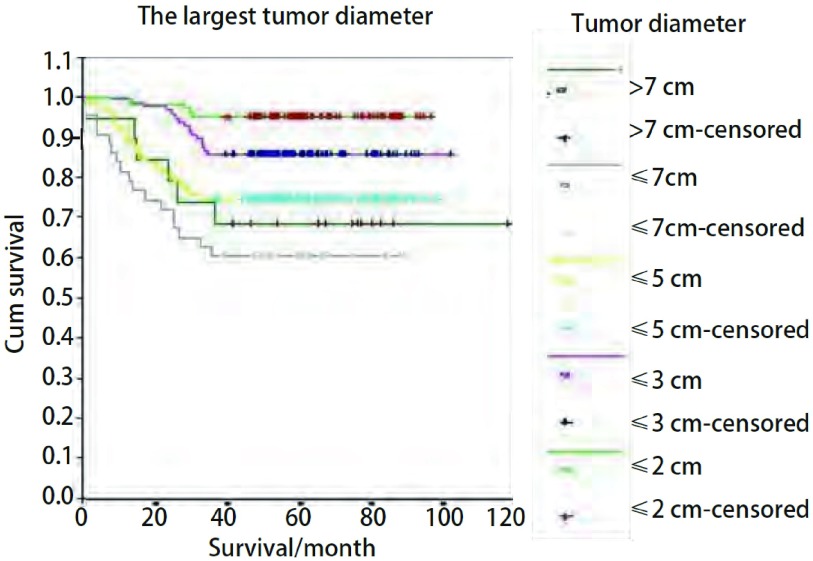
NSCLC病例基于肿瘤最长径的生存曲线(*P* < 0.000, 1) *Kaplan-Meier* survival curves based on tumor diameter in patients with NSCLC (*P* < 0.000, 1)

女性患者3年生存率优于男性(89.22% *vs* 77.53%, *P*=0.001, 8)。无淋巴管癌栓患者3年生存率优于淋巴管癌栓患者(82.46% *vs* 63.64%, *P*=0.073, 8)。无血管癌栓患者3年生存率优于血管癌栓患者(82.35% *vs* 62.5%, *P*=0.110, 0)。老年患者3年生存率较低(70.64% *vs* 85.85%, *P*=0.000, 1)。吸烟年支≥400患者3年生存率较低(77.59% *vs* 85%, *P*=0.035, 2)。多因素分析显示对生存期有影响的变量为年龄、性别、血管癌栓、组织学类型、脏层胸膜侵犯(表 2)。

**2:** 影响整体生存期的多因素*COX*回归模型 Multivariate *cox* proportional hazards model analyses of various factors affecting overall survival

Multivariate	*n*	HR (95%CI)	*P*
Age (yr)			
< 70	324		0.020
≥70	109	0.720 (0.546-0.949)	
Vascular invasion			
No	425		0.015
Yes	8	0.391 (0.183-0.832)	
Histology			
Adenocarcinoma	278		0.020
Squamous cell carcinoma	98	0.848 (0.314-2.291)	
Adenosquamous cell carcinoma	50	0.705 (0.254-1.959)	
Large-cell carcinoma	3	1.263 (0.448-3.566)	
Other types	4	0.531 (0.059-4.753)	
Visceral pleural involvement			
No	102		0.004
Yes	331	0.658 (0.495-0.874)	
Gender			
Female	167		0.001
Male	266	0.654 (0.505-0.847)	
HR:hazard ratio; CI:confidence interval.			

### 2.3 化疗对生存期的亚组分析

在全组分析中, 进行辅助化疗≥1周期患者共181例, 未化疗患者252例, 3年生存率化疗组更优(87.29% *vs* 78.26%, *P*=0.013, 3, 图 2)。Ⅰb期患者中, 进行辅助化疗≥1周期患者共142例, 未化疗患者167例, 两组3年生存率的差异无统计学意义(88.739% *vs* 82.63%, *P*=0.119, 2)。

**2:**
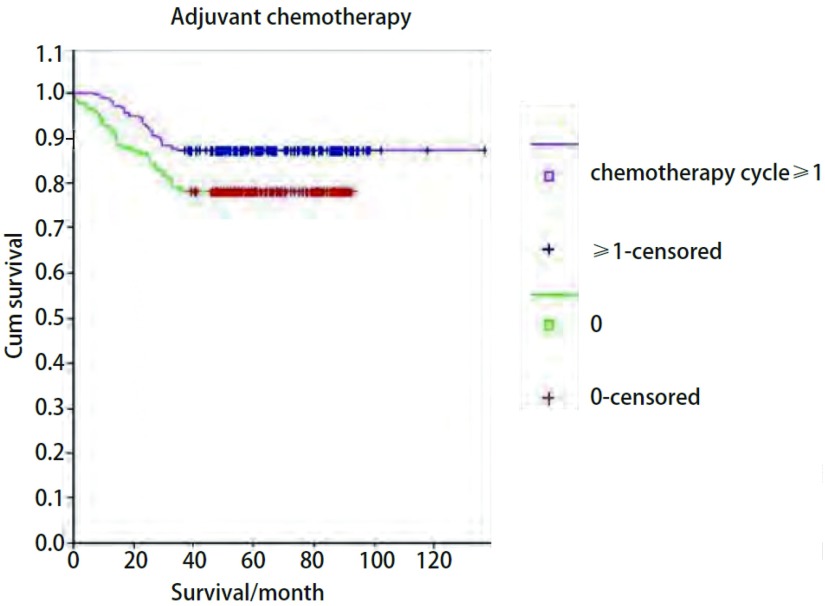
化疗组和未化疗组的生存曲线(*P*=0.013, 3) *Kaplan-Meier* survival curves between patients with chemotherapy and without chemotherapy (*P*=0.013, 3)

Ⅱa期/Ⅱb期患者中化疗组生存获益与未化疗组相比差异无统计学意义。胸膜受侵患者中化疗组3年生存率优于未化疗组(85.99% *vs* 76.76%, *P*=0.026, 5)。腺癌伴有混合亚型和腺鳞癌患者中化疗组的3年生存率与未化疗组相比差异无统计学意义(87.01% *vs* 76%, *P*=0.065, 9)。

## 3 讨论

本研究是一项回顾性分析, 时间从1998年6月-2010年6月, 时间跨度较大, 时间可能是一个较大的混杂因素。前期(2000年前)手术的患者因技术限制、器材条件等原因手术创伤大, 化疗方案副作用大(少数3药化疗), 且无后续表皮生长因子受体酪氨酸激酶抑制剂(epidermal growth factor receptor-tyrosine kinase inhibitor, EGFR-TKI)治疗, 这些都可能对生存有影响, 但本文未加讨论。因为后期手术例数较多, 无法与前期患者作对照分析。

本研究的患者中位生存时间未达到5年, 这可能是因为本研究中位随访时间仅为59.85个月, 延长随访时间可能会增加5年生存率。在未来的研究报告中会统计该群早期患者的5年生存率。本研究未纳入纵隔淋巴结清扫术中发现淋巴结转移的患者, 故不涉及新分期N1的患者。由于本研究的Ⅱ期(Ⅱa和Ⅱb)患者样本数少, 对Ⅱ期患者的术后预后因素分析还需更大样本的研究。

### 3.1 肿瘤最长径

1997年的肺癌分期系统在过去的十年中对规范肺癌的诊治及临床研究起到了很重要的作用, 但是由于样本量相对较小, 而且被用于研究的病例资料主要来源于同医疗机构, 其数据不具广泛性和代表性, 并且缺乏内部和外部的评价标准。随着各种先进检查手段的应用, 对肺癌临床分期判断的准确性也在不断地提高, 使老的分期方法渐渐显露出其不足之处。因此, IASLC于2007年在世界肺癌大会上提出了对第7版肺癌TNM分期的修改建议。基于*Log-rank*分析确定的最佳切入点, IASLC对肺癌的分期结果显T1肿瘤应分为2个亚组。因此, T1期肺癌分为了2个不同预后组:≤2 cm(T1a)和 > 2 cm但≤3 cm(T1b)。在符合手术完全切除、病理分期为N0 (R0 pN0)的患者中, 按照第6版肺癌分期标准并根据肿瘤大小进行调整, p T1a (肿瘤最大直径≤2 cm, *n*=1, 816)、p T1b(3 cm≥肿瘤最大直径 > 2 cm, *n*=1, 653)、p T2a (5 cm≥肿瘤最大直径 > 3 cm, *n*=2, 822)、p T2b(7 cm≥肿瘤最大直径 > 5 cm, *n*=825)、p T2c (肿瘤最大直径 > 7 cm, *n*=364);中位生存期分别为尚未到达、113个月、81个月、56个月和29个月; 5年生存率分别为77%、71%、58%、49%和35%, 各组间差异均有统计学意义(*P* < 0.05)^[11]^。本研究中T分期分析结果有充分确实的信息证明第7版肺癌TNM分期的建议值得采纳, 确认了以3 cm为界点来分类肿瘤大小是有效可行的。

### 3.2 性别与吸烟因素

过去对于肺癌生存性别差异的研究表明女性有生存优势。本研究确认了在早期肺癌术后患者中, 女性较男性的生存优势有统计学意义。多项研究^[12-16]^报道了术后NSCLC患者生存期有性别差异, 女性有更佳的生存结局, 女性并且淋巴结播散更少的患者生存率更好。外源性或内源性雌激素类基因、情绪因素也可能对女性患者肺癌的发展和生存起重要作用^[17, 18]^。

研究^[19]^证实大约70%的NSCLC患者存在EGFR的过表达, 并且这种过表达与患者的预后相关。在吸烟引发的NSCLC中, 约80%患者*EGFR*酪氨酸激酶区域的突变(包括缺失、插入和点突变)通常是外显子18、外显子19的框移突变和外显子21的点突变。因此, 临床可运用EGFR-TKI治疗晚期NSCLC。研究^[20, 21]^证实非吸烟、腺癌、亚洲东方女性患者对EGFR酪氨酸激酶抑制剂的治疗效果更加明显, 并且预后较好。同时, 非吸烟患者中EGFR的变异率为31%, 吸烟患者中*EGFR*的变异率为21%, 两者相比前者*EGFR*在外显子19区域的框移突变(可达60%)和外显子21区域(L858R)的错义突变(可达35%)可能性要高于后者。由此, 可以推论*EGFR*变异的频率与群体吸烟的数量和持续时间呈负相关。随着吸烟量和吸烟年数的增加, 外显子19和21区域突变的可能性会下降, 患者对于EGFR-TKI的敏感性也会下降。本研究中非吸烟和女性人群预后更好, 可能与该人群*EGFR*突变概率更高, 术后肺癌复发EGFR-TKI疗效更好, 从而使生存期延长有关。

### 3.3 化疗对生存期的亚组分析

在肿瘤完全切除的NSCLC患者中, 辅助化疗以已被证实能够改善早期肺癌患者的生存^[22-24]^。2004年在美国临床肿瘤年会上报道美国CALGB 9633实验组观察了344例Ⅰb患者完全切除术后辅助卡铂加紫杉醇化疗, 不加辅助放疗, 最长随访4年, 平均随访34个月, 生存率增加12%(*P* < 0.028)^[25]^。相对于大多数肺癌术后化疗研究的阴性结果, 这项研究令人振奋。但到了2006年美国临床肿瘤年会, 该研究^[26]^第5年的随访资料显示研究组与对照组的生存曲线又逐渐并到一起(5年生存率60% *vs* 57%, *P*=0.32), 这使得Ⅰ期NSCLC术后辅助化疗的作用再次成为不确定因素, 进一步的分层分析发现术后辅助化疗仅对肿瘤≥4 cm的Ⅰb期病变有临床意义。

2007年, 美国和加拿大两大肿瘤研究中心的研究报告提出了完全切除的NSCLC的术后治疗指导方针^[27]^, 对Ⅱa期、Ⅱb期及Ⅲa期患者建议常规进行术后辅助铂类化疗, 而对于Ⅰb期患者尽管一些试验证实术后化疗可能有一定的益处, 但不建议常规使用, 对Ⅰa患者则不建议使用。

2011版NCCN提出NSCLC术后的高危因素包括低分化、包括神经内分泌瘤、侵犯脉管、楔形切除、肿瘤 > 4 cm、脏层胸膜受累、NX。Ⅰa期且切缘阴性的患者接受观察。Ⅰa期切缘阳性患者治疗首先再次手术切除, 其次是化放疗(2B类证据级别)或放疗(2B类)。T2abN0且切缘阴性的患者一般仅接受观察, 有以上高危特征的患者推荐行辅助化疗; T2abN0患者如切缘阳性应该接受再切除术加化疗或化放疗加化疗。对于手术切缘阴性的Ⅱ期病变, 推荐化疗(1类)加或不加放疗(放疗为3类)。如果Ⅱ期患者切缘阳性, 可选治疗方法包括再次手术切除加化疗或化放疗联合化疗^[28]^。

本研究中Ⅰb期患者309例, 3年生存率化疗组为88.73%, 未化疗组为82.63%, 但差异无统计学意义(*P*=0.119, 2)。这一结果符合指南推荐, 显示辅助化疗可能有益Ⅰb期患者生存, 但需要更大样本和更长随访时间的研究支持该结果。

腺癌是非吸烟患者中发生率最高的类型。基因表达谱检测(采用DNA微阵列)发现肺腺癌的亚型(即支气管腺癌、鳞腺癌、大细胞腺癌)与分期特异的生存期和转移模式有关。支气管腺癌与早期肺癌生存期延长有关^[29]^。本研究发现对腺癌伴有混合亚型和腺鳞癌患者, 其3年生存率化疗组与未化疗组相比差异无统计学意义(*P*=0.065, 9)。因此, Ⅰb期患者是否进行辅助化疗需要考虑其病理类型, 这需要进一步的研究加以确认。

虽然此次研究为回顾性研究, 但是为未来随机前瞻性研究的设计和治疗策略提供了重要的参考依据。在T分期方面得到了与IASLC相似的结果。第七版TNM分期有临床指导意义, Ⅰb期完全切除的患者如存在NCCN定义的高危因素, 术后辅助化疗能使患者得到更大的生存获益。
